# A one year longitudinal study of cortical myelination changes following pediatric mild traumatic brain injury^☆^

**DOI:** 10.1016/j.nicl.2025.103837

**Published:** 2025-06-30

**Authors:** Jessica R. McQuaid, Tracey V. Wick, Josef Ling, Andrew B. Dodd, Divyasree Sasi Kumar, Upasana Nathaniel, Samuel D. Miller, Vadim Zotev, Harm J. van der Horn, John P. Phillips, Richard A. Campbell, Robert E. Sapien, Timothy B. Meier, Andrew R. Mayer

**Affiliations:** a The Mind Research Network/Lovelace Biomedical Research Institute, Albuquerque, NM 87106, United States; b University of Groningen, University Medical Center Groningen, Groningen, the Netherlands; c Departments of Neurology, University of New Mexico, Albuquerque, NM 87131, United States; d Psychiatry and Behavioral Sciences, University of New Mexico, Albuquerque, NM 87131, United States; e Emergency Medicine, University of New Mexico, Albuquerque, NM 87131, United States; f Departments of Psychology, University of New Mexico, Albuquerque, NM 87131, United States; g Departments of Neurosurgery, Medical College of Wisconsin, Milwaukee, WI 53226, United States; h Departments of Cell Biology, Neurobiology and Anatomy, Medical College of Wisconsin, Milwaukee, WI 53226, United States; i Departments of Biomedical Engineering, Medical College of Wisconsin, Milwaukee, WI 53226, United States

**Keywords:** Pediatric mild traumatic brain injury, Cortical, Myelin, Neurodevelopment

## Abstract

The impact of pediatric mild traumatic brain injury (pmTBI) on cortical (i.e., grey matter) myelination is not yet understood, especially for interactions with neurodevelopment. The current study examined the impact of pmTBI on cortical myelination relative to healthy controls (HC) by estimating myelin content using the T_1_w/T_2_w ratio method. Data were obtained from pmTBI (*N* = 217) participants at approximately 7 days (Visit 1 [V1]), 4 months (Visit 2 [V2]), and 1 year (Visit 3 [V3]) post-injury, with equivalent sampling points for age and sex-matched HC (*N* = 180). Clinical results suggested only partial recovery from post-concussive symptoms from V1 to V3, with similar incomplete recovery of sleep, functional outcomes, behavior, and long-term memory. Myelin content increased with chronological age and as a function of individual aging across study visits in a hemisphere specific fashion (left > right), most visibly within the posterior parietal lobe. Myelin content was also greater for females relative to males. There was evidence of both a reduction in myelination within the posterior parietal cortex for the pmTBI group at 4 months post-injury, as well as evidence of increased myelination within the left prefrontal cortex at one-year post-injury. However, neither of these findings survived various sensitivity analyses, suggesting that there were minimal effects of pmTBI on cortical myelin content in general. In summary, although rapid changes in myelin content existed as a function of neurodevelopment, there was little evidence to suggest that pmTBI permanently altered cortical myelin development trajectories.

## Highlights

• Pediatric mild traumatic brain injury has little to no effect on myelination.
• Developmental myelination has a strong correlation with age.
• Changes in myelination were greater in the left relative to right hemisphere.

## 1 Introduction

Late childhood and adolescence mark a time of significant physical, emotional, and cognitive change (Corrigan et al., 2021, Spear, 2013). Neurodevelopment during this critical period is primarily characterized by synaptic pruning, cortical thinning, and widespread increases in cortical myelination (Corrigan et al., 2021, Kwon et al., 2020, Tamnes et al., 2017), all of which can be adversely affected by pediatric “mild” traumatic brain injury (pmTBI; used synonymously herein with concussion). Recent evidence suggests that physiological recovery may continue for months following pmTBI (Kamins et al., 2017), regardless of clinical status. Preclinical studies have reported decreased myelin content based on histology and imaging findings (Mierzwa et al., 2015; Molina et al., 2020). To date, most clinical studies have focused on white matter (WM) rather than cortical myelination and have been conducted in collegiate or adult cohorts (Jurick et al., 2018, Russell-Schulz et al., 2021, Spader et al., 2018, Wright et al., 2016). WM myelination has been shown to decrease post-injury (Wright et al., 2016) but return to baseline (Jurick et al., 2018, Wright et al., 2016) in adults. Other studies have suggested evidence of remyelination (Spader et al., 2018) or persisting demyelination into more chronic stages post-injury in adult mTBI (Russell-Schulz et al., 2021, Yoo et al., 2022). One adult study showed evidence of more rapid cortical demyelination following mTBI (Mahoney et al., 2022). To our knowledge, the short- and long-term effects of pmTBI on grey matter (GM) myelin content and neurodevelopment remain relatively understudied (Mayer et al., 2018).

The rate of GM myelination during neurodevelopment (i.e. spanning many years) is non-linear, with myelination occurring first in motor and sensory cortices and heteromodal cortical areas remaining more sparsely myelinated in younger children (Glasser et al., 2014, Gogtay and Thompson, 2010, Lenroot and Giedd, 2006, Shafee et al., 2015, Spear, 2013, Timmler and Simons, 2019). A higher correlation exists between age and GM (cortical) myelination relative to WM, leading to the suggestion that GM myelination plays a more significant role in the development of higher-order cognitive processes during adolescence than previously understood (Corrigan et al., 2021). Myelination is the greatest within the posterior parietal lobule during late childhood and adolescence (Corrigan et al., 2021, Grydeland et al., 2019, Sydnor et al., 2021, Tamnes et al., 2017). Although multiple measures exist for quantifying myelin content *in vivo*, it is most typically inferred from diffusion studies despite the known rapid relaxation of myelin water between sheaths (Corrigan et al., 2021, Lazari and Lipp, 2021, MacKay and Laule, 2016, Shafee et al., 2015, Shams et al., 2019, van der Weijden et al., 2021). The T_1_w/T_2_w ratio method has more recently been used for *in vivo* quantification (Glasser et al., 2014, Glasser and Van Essen, 2011), with strong agreement to other GM myelin MRI estimation methods (Hagiwara et al., 2018, Shams et al., 2019), histology (Glasser and Van Essen, 2011), as well as robustness to movement artifacts (Shams et al., 2019).

The current study therefore examined GM myelination changes in large cohorts of pmTBI (*N* = 217) and age- and sex-matched healthy controls (HC; *N* = 180) approximately 7 days (Visit 1 [V1]), 4 months (Visit 2 [V2]) and 1 year (Visit 3 [V3]) post-injury using the T_1_w/T_2_w method. Our primary hypothesis was that GM myelin content would decrease following pmTBI, with evidence for partial recovery at 4- and 12-months post-injury. Given the rapid changes that occur during this critical neurodevelopmental period, we predicted that trauma-related changes in myelin content would be most prominent within the posterior parietal cortex. A secondary hypothesis was that any changes in myelin content would be associated with injury severity as indexed by the presence of loss of consciousness (LOC) and post-traumatic amnesia (PTA) (Mayer et al., 2023). In contrast, no association was expected between myelin content and post-concussive symptom (PCS) due to the relatively poor psychometric properties and non-specific nature of symptom self-report (Iverson et al., 2015, Mayer et al., 2023).

## 2 Methods

### 2.1 Participants

247 patients with pmTBI (8–18 years) were consecutively recruited from local emergency departments and urgent care clinics from July 2016 to December 2022. Patients were diagnosed by clinicians independent of the study, and subsequently confirmed by the senior author. Inclusion criteria are represented by a combination of the original American Congress of Rehabilitation Medicine (upper limits: Glasgow Coma Scores ≥ 13, maximum loss of consciousness [LOC] = 30 min, and maximum post-traumatic amnesia [PTA] = 24 h) and Zurich Concussion in Sport Group (lower limits: at least two new symptoms) guidelines. 200 sex- and age-matched HCs were recruited from the local community through fliers and word of mouth, and evaluated at equivalent time points. All participants provided informed consent or assent according to institutional guidelines by the University of New Mexico Health Sciences’ Human Research Review Committee (HRRC).

Both pmTBI and HC were excluded for history of major neurological diagnoses, moderate or severe TBI (>30 min LOC), history of recent concussion (less than 6 months) other than the referring incident, major developmental disorders (autism spectrum disorder or intellectual disability), psychiatric disorders other than adjustment disorder, substance abuse/dependence, non-English fluency, or contraindications to MRI. Additional exclusion criteria for HC included attention-deficit/hyperactivity disorder or a learning disability. Urine-based drug screens were conducted for all participants at all visits, and individuals with positive tests were excluded except for recreational marijuana use. One HC participant was disenrolled from the study between V1 and V2 due to non-compliance with appointment scheduling and attendance.

**Fig. 1:**
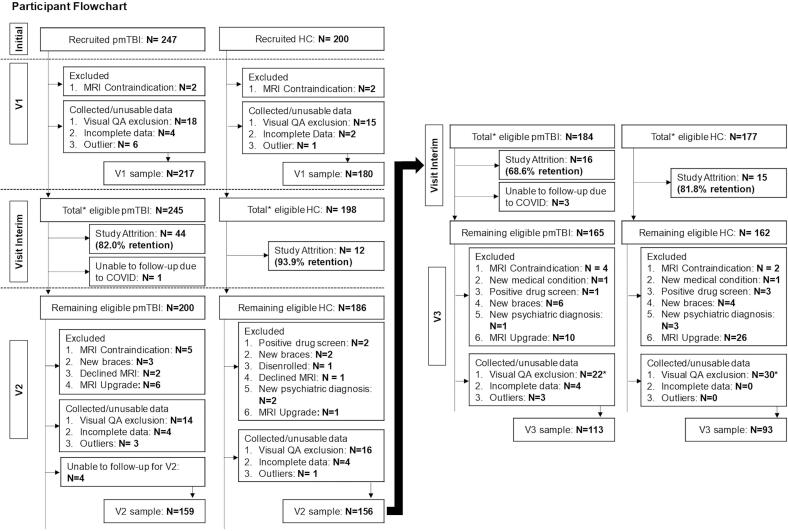
Participant Flowchart. Flowchart of participant enrollment, inclusion, data quality assurance, and attrition from Visit 1 (V1), V2, and V3 used in the analyses of participants with pediatric mild traumatic brain injury (pmTBI) and healthy controls (HC). There were several participants (5 pmTBI and 13 HC) who exhibited poor T_2_ quality due to a scanner software upgrade. This increased the amount of visual QA exclusions (22 pmTBI and 30 HC total) as denoted by the asterisk.

The final sample number following all quality assurance steps and outlier analyses (Supplemental Methods) included a total of 217 pmTBI (91 females; 14.3 ± 2.9 years old) along with 180 sex- and age-matched HC (85 females; 14.2 ± 2.9 years old) at V1. Follow-up analyses included a total of 159 pmTBI (69 females; 14.5 ± 2.8 years old) along with 156 HC (69 females; 14.3 ± 2.9 years old) at V2, with 113 pmTBI (51 females; 15.2 ± 3.0 years old) along with 93 HC (43 females; 14.8 ± 3.1 years old) at V3. See Fig. 1 and Supplemental Methods for full details on enrollment, quality assurance, and retention rates.

### 2.2 Clinical assessments

A Common Data Elements battery of neuropsychological tests and questionnaires were administered at all visits to evaluate PCS, emotional and cognitive functioning, behavioral issues, quality of life, reading ability and effort, and to investigate cognitive deficits (attention, processing speed, executive functioning, working memory, and long-term memory). See Supplemental Methods and Supplemental Table S1 for full details. A normative rather than simple change approach was used to binarily define individuals with high PCS burden (i.e., symptomatic versus asymptomatic) and reduce false positive rates (Mayer et al., 2020). A semi-structured pediatric interview was used to establish a previous history of TBI (Hergert et al., 2022).

### 2.3 Image acquisition and processing

Imaging data were collected on a 3T Siemens Tim Trio MRI scanner with a 32-channel head coil (321 participants; 71.8%) or a 3T Siemens Prisma Fit scanner (126 participants, 28.2%). Imaging data were not collected for 43 participants (16 pmTBI; 27 HC) at V2 and V3 due to the scanner upgrade, and known differences in the gradient set. All T_1_-weighted (T_1_w) data were collected with similar multi-echo sequences across both scanner platforms (1 mm^3^ voxels), whereas T_2_-weighted (T_2_w) sequences varied across the Trio (1.1 × 1.1 × 1.5 mm voxel; 2D T_2_ TSE) and Prisma Fit (1 mm^3^ voxels; 3D T_2_-SPACE) platforms (Supplemental Methods). Scanner platform was therefore included as a covariate in all analyses (Supplementary Fig. S4). All structural images were reviewed by a board-certified neuroradiologist blinded to participant diagnosis.

**Fig. 2:**
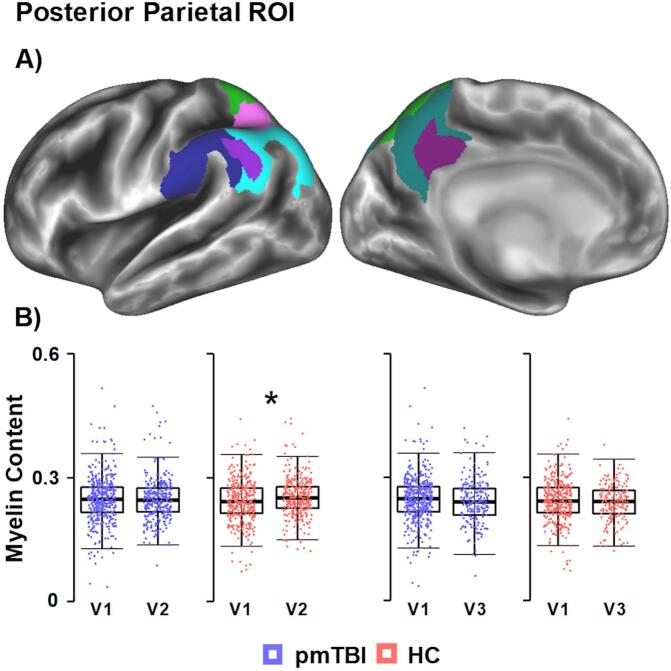
Posterior Parietal Region of Interest (ROI) Results. Panel A displays seven regions within the posterior parietal lobe (angular gyrus, supramarginal gyrus, superior parietal lobule, precuneus, sulcus intermedius primus, intraparietal sulcus and transverse parietal sulci, and the subparietal sulcus) which were selected from the Destrieux atlas. Panel B plots data from both participants with pediatric mild traumatic brain injury (pmTBI) and healthy controls (HC) for Visit 1 (V1), V2 and V3. The asterisk indicates that the HC had increased myelin content at V2 compared to V1 (i.e., within-subject), but there were no other significant group differences. Moreover, the Group × Visit findings were no longer significant following additional sensitivity analyses with the ComBat algorithm.

T_1_w and T_2_w images were analyzed using the Human Connectome Project (HCP) processing pipeline as previously described (Glasser et al., 2013). Briefly, the PreFreeSurfer pipeline registered the T_2_w volume to T_1_w space, applied a bias field correction (B1) to minimize distortion, and aligned the subject’s structural space to MNI space. The FreeSurfer pipeline then segmented volumes into predefined structures, reconstructed white, and pial cortical surfaces, performed standard folding-based surface registration to surface atlas, and was smoothed across the surface (FWHM = 6 mm). The PostFreeSurfer pipeline produced high-resolution, smoothed myelin maps in GIFTI format. Visual quality assurance was completed by a minimum of two trained research staff for all visits and participants. A priori parietal ROI analyses were conducted with the Destrieux Atlas on FreeSurfer v.7.1.1 focusing on a weighted average using the area of seven parcellations (Fig. 2 Supplemental Table S2) from the posterior parietal cortex (Grydeland et al., 2019, Sydnor et al., 2021).

### 2.4 Statistical analyses

All demographic and clinical analyses were conducted using SPSS (IBM Corp. Released 2011. IBM SPSS Statistics for Windows, Version 20.0. Armonk, NY: IBM Corp) using either general estimating equations (GEE) or generalized linear models (GLM). Gamma, negative binomial, or Gaussian distributions were determined using information criterion results, and clinical results were Bonferroni-corrected for primary versus secondary measures (Supplemental Methods). All analyses were conducted using 2 × 2 models that separately compared V1 to either V2 or V3 [e.g., Group (pmTBI v. HC) × Visit (V1 v. other)] and included age (unit = month) as a covariate. The latter examined for neurodevelopmental effects and modeled potential effects associated with age-at-injury (i.e., Age × Group interactions). Whole-brain vertex-wise (AFNI’s 3dLME) and ROI (SPSS) results were completed using similar 2 × 2 (Group × Visit) linear mixed effects (LME) models. Supplemental 2 × 2 × 2 [Group (pmTBI v. HC) × Visit (V1 v. other) × Sex (male v. female)] LME models examined the potential moderating effects of sex on cortical myelination differences in pmTBI with self-reported pubertal status included as an additional covariate. Age was not included in this Supplemental model given the expected collinearity with self-reported pubertal stage. Scanner platform was used as a nuisance variable for all analyses, and ROI analyses included hemisphere as an additional factor. Finally, sensitivity analyses were conducted for all primary clinical and imaging data to ensure attrition did not unduly affect results (Supplemental Methods). Similarly, additional sensitivity analyses were performed for all primary imaging analyses to examine for the effects of data harmonization across scanner platforms using the ComBat algorithm (Fortin et al., 2018).

All clinical results were classified based on patterns of complete recovery (i.e., Group × Visit interaction with non-significant Group effects at V2/V3), partial recovery (i.e., Group × Visit interaction with smaller but significant Group effects at V2/V3), a mixed pattern (i.e., Group × Visit interaction at V1/V2 but significant group effect between V1/V3), no evidence of recovery (i.e., main effect of Group), or no evidence of deficits (i.e., absence of main effects or interaction).

## 3 Results

### 3.1 Demographics

Groups did not differ on biological sex, age, self-reported Tanner stage of development, or handedness (all *p*’s ≥ 0.05; Supplemental Table S3) at V1. Significant group differences were observed for self-reported history of previous head injuries (*χ^2^* = 11.93, *p* = 0.008; pmTBI = 18.0%, HC = 7.3%), parental self-reported psychopathology (*Wald-χ^2^* = 23.36; *p <* 0.001; pmTBI > HC), premorbid reading ability (*Wald-χ^2^* = 47.37; *p* < 0.001; pmTBI < HC) and effort (*Wald-χ^2^* = 38.49; *p* < 0.001; pmTBI < HC). Reading ability and effort were therefore used as covariates during neuropsychological analyses. Eleven pmTBI participants (9.5%) had a positive CT scan from the 116 CT scans acquired as part of routine care. A small but significant difference for days between visits (DBV) between V1 and V2 (mean difference = 4.49 days) was observed (*p* = 0.021; HC > pmTBI) between groups with no significant differences between V1 and V3.

### 3.2 Clinical outcomes

Table 1 presents central tendency data for clinical and neuropsychological results. Primary clinical variables (see Table 2 for associated statistics and recovery patterns) indicated a partial recovery of PCS burden (i.e., significant but smaller effects observed at V2 and V3 relative to V1) with 38.2% symptomatic at V1, 15.7% symptomatic at V2 and 16.8% symptomatic at V3. There was no evidence of deficits in quality of life or self-reported behavioral disturbances. Secondary clinical measures indicated a complete recovery of pain and headache following V1. Although self-reported anxiety appeared to be recovered at V2, evidence of increased anxiety (pmTBI > HC) was again observed at V3. Continued disturbances in sleep, global outcomes (GOS-E), and behavioral and emotional difficulties (SDQ) were observed across all visits. Finally, there was no difference in self-reported depression at any of the visits.

**Table 1:** Clinical and Neuropsychological Data.

Metric	V1 pmTBI	V1 HC	V2 pmTBI	V2 HC	V3 pmTBI	V3 HC
Symptom Measures						
PCSI (% Max) (P)	16.7 (5.6–37.3)	3.2 (0.2–8.8)	4.0 (0.8–14.3)	4.8 (0.8–9.3)	4.8 (0.8–15.9)	2.9 (0.0–8.8)
PROMIS Sleep (S)	18.9 ± 7.1	14.3 ± 4.8	18.0 ± 6.9	15.2 ± 4.9	18.5 ± 6.1	14.9 ± 5.0
PROMIS Anxiety (S)	2.0 (0.0–7.0)	1.0 (0.0–4.0)	2.0 (0.0–7.0)	2.0 (0.0–5.0)	2.0 (0.0–6.0)	0.5 (0.0–4.0)
PROMIS Depression (S)	2.0 (0.0–10.0)	1.0 (0.0–4.0)	1.0 (0.0–6.0)	1.0 (0.0–5.0)	1.0 (0.0–6.0)	0.5 (0.0–4.0)
Pain Scale (S)	3.0 (1.0–5.0)	0.0 (0.0–1.0)	0.0 (0.0–3.0)	0.0 (0.0–1.0)	0.0 (0.0–2.0)	0.0 (0.0–1.0)
HIT-6 (S)	52.0 (43.0–60.0)	40.0 (36.0–46.0)	44.0 (40.0–54.0)	42.0 (38.0–48.0)	46.0 (40.0–52.0)	41.0 (38.0–46.0)
Behavioral & Outcome Measures						
CBQ (P)	1.0 (0.0–3.0)	1.0 (0.0–2.0)	1.0 (0.0–3.0)	1.0 (0.0–2.0)	1.0 (0.0–3.0)	0.0 (0.0–2.0)
PedsQL (P)	N/A	N/A	83.4 ± 13.9	86.7 ± 9.8	85.1 ± 12.1	88.9 ± 9.4
SDQ (S)	N/A	N/A	6.0 (4.0–10.0)	4.0 (2.0–7.0)	6.0 (3.0–9.0)	4.0 (2.0–6.0)
GOS-E (S)	1.0 (1.0–4.0)	1.0 (1.0–1.0)	1.0 (1.0–2.0)	1.0 (1.0–1.0)	1.0 (1.0–1.0)	1.0 (1.0–1.0)
TOMMe10 (S)	10.0 (9.0–10.0)	10.0 (10.0–10.0)	10.0 (10.0–10.0)	10.0 (10.0–10.0)	10.0 (10.0–10.0)	10.0 (10.0–10.0)
WRAT-IV (S)	49.6 ± 10.1	56.8 ± 10.6	51.2 ± 11.1	58.5 ± 11.3	52.0 ± 10.5	57.3 ± 11.3
PS (P)	46.6 ± 7.9	51.0 ± 8.5	50.5 ± 8.6	53.0 ± 9.2	52.3 ± 8.4	55.5 ± 9.9
AT (P)	46.8 ± 9.0	51.8 ± 6.8	50.1 ± 7.7	52.1 ± 7.6	50.4 ± 7.1	52.3 ± 7.9
WM* (S)	46.9 ± 8.2	51.0 ± 9.9	47.5 ± 9.7	51.6 ± 11.0	48.4 ± 8.9	51.6 ± 10.0
EF (S)	47.0 ± 7.5	51.4 ± 6.3	50.6 ± 6.8	53.4 ± 6.4	53.0 ± 6.2	54.5 ± 6.7
HVLT Delayed Recall (S)	7.4 ± 2.7	8.7 ± 2.0	7.4 ± 2.2	8.4 ± 2.4	8.3 ± 2.4	9.5 ± 2.0

Notes: V1 = Visit 1 (∼7 days post-injury); V2 = Visit 2 (∼4 months post-injury); V3 = Visit 3 (∼1 year post-injury); HC = healthy control; pmTBI = pediatric mild traumatic brain injury; PCSI= Post-Concussion Symptom Inventory (presented as percent of maximum score to account for age-related scale differences); PROMIS= Patient Reported Outcomes Measurement Information System; HIT-6=Headache Impact Test; CBQ= Conflict Behavior Questionnaire; SDQ= Strengths and Difficulties Questionnaire; PedsQL= Pediatric Quality of Life Inventory; GOS-E = Glasgow Outcome Scale Extended; TOMMe10 = Test of Memory Malingering – 10-item short version; WRAT-IV = Wide Range Achievement Test 4; PS = processing speed; AT = attention; WM = working memory; EF = executive function; HVLT Delay = Delayed recall on Hopkins Verbal Learning Task (measure of long-term memory). Data are either formatted at mean ± standard deviation or median (interquartile range).

**Table 2:** Statistics for Clinical Data.

Pattern	Measure	Model V1 vs. V2			Sensitivity (*p*-value)		Model V1 vs. V3			Sensitivity (*p*-value)	
		Effect (*Wald-χ^2^*; *p-*value)	V1 (*p-*value)	V2 (*p-*value)	pmTBI	HC	Effect (*Wald-χ^2^*; *p-*value)	V1 (*p-*value)	V3 (*p-*value)	pmTBI	HC
Complete Recovery	Pain Scale (S)	G × V (*χ2 = 15.61; p < 0.001)*	<0.001	0.012*	0.935	0.267	G × V (*χ2 = 8.34; p = 0.004)*	<0.001	0.086	0.981	0.362
	Headache (S)	G × V (*χ2 = 42.08; p < 0.001)*	<0.001	0.279	0.266	0.071	G × V (*χ2 = 23.82; p < 0.001)*	<0.001	0.069	0.801	0.289
	Attention (P)	G × V (*χ2 = 15.39; p < 0.001)*	0.001	0.725	0.488	0.021	G × V (*χ2 = 10.42; p = 0.001)*	0.001	0.862	0.187	0.026
	EF (S)	G × V (*χ2 = 6.03; p = 0.014)*	0.003	0.297	0.567	0.169	G × V (*χ2 = 6.63; p = 0.010)*	0.003	0.843	0.730	0.385
Partial Recovery	PCS (P)	G × V (*χ2 = 34.21; p < 0.001)*	<0.001	0.003	0.452	0.941	G × V (*χ2 = 17.40; p < 0.001)*	<0.001	0.002	0.573	0.614
Mixed Recovery	Anxiety (S)	G × V (*χ2 = 7.53; p = 0.006)*	<0.001	0.185	0.392	0.489	G (*χ2 = 15.41; p < 0.001)*			0.391	0.631
	PS (P)	G × V (*χ2 = 6.78; p = 0.009)*	0.004	0.429	0.574	0.863	G (*χ2 = 12.07; p = 0.001)*			0.413	0.182
No Recovery	Sleep (S)	G (*χ2 = 29.82; p < 0.001)*			0.233	0.681	G (*χ2 = 30.88; p < 0.001)*			0.963	0.216
	Global Outcome(S)	G (*χ2 = 27.72; p < 0.001)*			0.738	0.973	G (*χ2 = 23.20; p < 0.001)*			0.285	0.928
	Behavioral& Emotional Difficulties(S)	G (*χ2 = 9.47; p = 0.002)*			0.436	0.503	G (*χ2 = 8.49; p = 0.004)*			0.012	0.312
	Long-Term Memory (S)	G (*χ2 = 13.64; p < 0.001)*			0.800	0.903	G (*χ2 = 19.72; p < 0.001)*			0.432	0.462
No Deficits	Depression (S)				0.667	0.420				0.541	0.437
	WM (S)				0.455	0.943				0.353	0.459
	PedQL(P)				0.154	0.891				0.109	0.255
	Behavioral Disturbance (P)				0.115	0.766				0.071	0.265

Note: (P): Primary domain; (S): Secondary domain; G: Group; V1/V2/V3: Visit 1/2/3 respectively; EF: executive function; PCS: post-concussive symptoms; PS: processing speed; WM: working memory; PedQL: Pediatric Quality of Life. All significant main effects of Group exhibited opposite patterns for clinical (pmTBI > HC) relative to neuropsychological (pmTBI < HC) findings. *denotes p-value not significant following Bonferroni correction. Complete recovery: V1 and V2 deficits with recovery by V3; Partial recovery: significant but smaller effects observed at V2 and V3 relative to V1; Mixed recovery: V2 recovery and V3 deficits (i.e., main effect of Group); No recovery: Deficits observed at all visits (i.e. main effect of Group); No Deficits: No significant main effects or interactions associated with Group.

Primary neuropsychological domains indicated complete recovery for attention following V1, with a mixed pattern of V2 recovery and V3 deficits (i.e., main effect of Group) for processing speed. Secondary cognitive measures indicated a complete recovery for executive function following V1 versus continued deficits in long-term memory across all visits in the pmTBI cohort. Finally, there was no evidence of deficits in working memory at any of the visits.

Sensitivity analyses (uncorrected for 30 comparisons per Group) comparing returning and non-returning participants were negative for all clinical and cognitive measures except for attention (HC only) as well as behavioral and emotional difficulties (pmTBI V3 only).

### 3.3 Cortical myelin Results: V1 relative to V2

Whole-brain vertex-wise results were null for the main effect of Group and all Group interactions when comparing ∼7 days (V1) and ∼4 months (V2) post-injury cortical myelin content following corrections for family-wise error. Significant main effects were observed for Visit in a hemisphere-specific fashion (left hemisphere [LH] > right hemisphere [RH]; Supplementary Fig. S1), with cortical myelination significantly increasing across both groups in predominantly non-frontal, LH regions across the relatively short 4-month interscan interval. There was a notable lack of visit effects within the left central sulcus. Widespread positive associations also existed between age and myelin throughout the bilateral cortical mantle (Supplementary Fig. S2) with the exception of the prefrontal cortex. The strongest relationships with age were observed in the precentral gyrus, postcentral gyrus, paracentral gyrus and sulcus, and superior parietal gyrus (Supplementary Fig. S2), with notably lower correlations present for the bilateral central sulcus. The inclusion of sex and self-reported pubertal status in a separate model (Supplementary Fig. S3) did not alter the null effects associated with Group or Group × Visit interaction.

ROI analyses for the posterior parietal cortex indicated significant Group × Visit *(F* = 4.16; *p* = 0.042), Visit × Hemisphere (*F* = 7.48; *p =* 0.007), Age × Hemisphere (*F* = 20.78; *p <* 0.001) and Group × Visit × Hemisphere × Age (*F* = 4.56; *p* = 0.033) interactions. Main effects of Visit (*F* = 12.92; *p <* 0.001; V1 < V2), Hemisphere (*F* = 490.46; *p <* 0.001; LH < RH), and Age (*F* = 156.03; *p <* 0.001) were also present. Follow-up analysis for the Group × Visit interaction yielded insignificant results when comparing HC to pmTBI at V1 (*p* = 0.501; Cohen’s d = -0.06) or V2 (*p* = 0.223; Cohen’s d = 0.13). However, the Group × Visit interaction indicated significant increases in myelin content for the HC at V2 (*p* < 0.001; all participants_d_ = 0.32; V1 < V2) but not for the pmTBI (*p* = 0.254; all participants_d_:=0.10) cohort (Fig. 2). Moreover, this effect was no longer present following additional sensitivity analyses to correct for scanner platform using the ComBat algorithm. Follow-up analysis for the Visit × Hemisphere interaction indicated a significant increase in LH myelin at V2 (*F* = 8.55; *p =* 0.004; all participants_d_ = 0.26; V1 < V2) but not for the RH (*F* = 2.52; *p =* 0.113; all participants_d_ = 0.15; V1 ∼ V2). Follow-up analysis of the Age × Hemisphere interaction indicated a significant effect of Age in both the LH (slope estimate = 0.009; *F* = 193.88; *p* < 0.001) and RH (slope estimate = 0.011; *F* = 235.27; *p* < 0.001).

### 3.4 Cortical myelin Results: V1 relative to V3

**Fig. 3:**
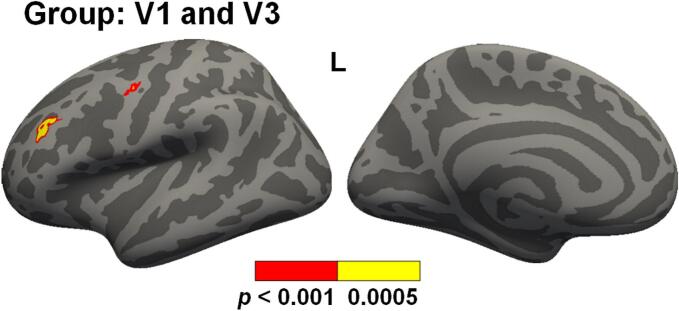
Vertex-wise cortical myelin group results. Two small regions within left (L) frontal middle gyrus and precentral gyrus showed significantly increased myelin content in participants with pediatric mild traumatic brain injury (pmTBI) relative to the healthy control (HC) group at Visits 1 (V1) and V3 (denoted by mean bar). Significant results are depicted for p < 0.001 (red) and p < 0.0005 (yellow) thresholds. The inclusion of sex in the model moderated the Group effect within the precentral gyrus (i.e., no longer significant). Moreover, neither finding was significant following additional sensitivity analyses with the ComBat algorithm.

Vertex-wise analyses comparing cortical myelin content at ∼7 days (V1) and ∼1 year (V3) post-injury indicated null results for Group × Visit × Age, Group × Age, and Group × Visit interactions. A significant main effect for Group was present in two small regions within the left middle frontal gyrus (156.68 mm^2^) and left precentral gyrus (44.47 mm^2^), indicating increased myelination for pmTBI at ∼ 1 year (Fig. 3). Significant effects of increased myelin concentrations as a function of study visit (V3 > V1) were observed in similarly predominantly LH regions as the 4-month comparison, with minimal visit effects present within the left central sulcus. However, decreased myelin content was observed within the left cingulate and intracingulate sulcus at V3 relative to V1 (Supplementary Fig. S1). Positive associations between age and cortical myelin content (Supplementary Fig. S2) was again observed across most of the bilateral cortical mantle in similar regions as the V1 relative to V2 results, although the magnitude of the correlation was slightly decreased within the paracentral gyrus and sulcus. The inclusion of sex and self-reported pubertal status in a separate model (Supplementary Fig. S3) resulted in the same finding of increased cortical myelination in the left middle frontal gyrus for pmTBI, whereas the Group effect within the precentral gyrus was no longer significant. The Group × Visit interaction remained null in this Supplemental model. However, findings of increased myelin content in the left prefrontal cortex were no longer significant following additional sensitivity analyses to correct for scanner platform using the ComBat algorithm.

ROI analyses for the posterior parietal cortex indicated a significant Visit × Hemisphere (*F* = 4.85; *p =* 0.029) and Age × Hemisphere (*F* = 27.39; *p <* 0.001) interaction, as well as main effects of Visit (*F* = 11.49; *p =* 0.001; V1 < V3), Hemisphere (*F* = 377.43; *p <* 0.001; LH < RH) and Age (*F* = 122.96; *p <* 0.001). However, follow-up analysis for the Visit × Hemisphere interaction indicated no significant differences in myelin content in either the LH (*F* = 2.69; *p =* 0.102; all participants_d_ = 0.41; V1 ∼ V3) or the RH (*F* = 0.07; *p =* 0.792; all participants_d_ = 0.25; V1 ∼ V3) between V1 and V3. Follow-up analysis of the Age × Hemisphere interaction indicated a significant effect of Age in both the LH (slope estimate = 0.008; *F* = 148.77; *p* < 0.001) and RH (slope estimate = 0.010; *F* = 182.20; *p* < 0.001).

Sensitivity analyses comparing returning and non-returning participants were negative for all voxelwise and ROI results relative to both V2 and V3.

### 3.5 Injury severity and relationships to cortical myelination

A GLM was conducted to examine a priori hypothesis regarding the relationship between traditional injury severity metrics (absence or presence of loss of consciousness/post-traumatic amnesia, mechanisms of injury [sports-related concussion versus other] and symptom burden [total PCSI score]) on findings of increased cortical myelination at V3. The analysis also controlled for scanner related effects as well as age and was limited to the pmTBI group only to avoid spurious correlations. A change score calculation (V3 – V1) was first calculated from the middle and precentral gyrus regions for each visit, followed by a weighted average. Results from the GLM were not significant for any of the injury severity mechanisms (*p* > 0.10).

## 4 Discussion

Myelination represents an important developmental aspect of late childhood and adolescence, contributing to neural connectivity and cognitive function (Corrigan et al., 2021, Kwon et al., 2020, Tamnes et al., 2017). Consistent with previous literature (Corrigan et al., 2021, Grydeland et al., 2019, Grydeland et al., 2013), myelin content increased with chronological age as well as a function of individual aging across study visits in a region-specific fashion. Importantly, significant increases in myelin content occurred as soon as 4 months across both pmTBI and HC. However, our primary hypothesis of reduced myelin content/altered neurodevelopmental trajectories post-pmTBI was generally not supported, with no significant differences in whole-brain cortical myelination between pmTBI and HC at 4 months or 1 year post-injury across multiple sensitivity analyses. Specifically, two small regions within the left middle frontal gyrus and left precentral gyrus exhibited contrasting evidence of increased myelination at 1-year post-injury, but these effects were not associated with common injury severity metrics and were no longer significant during additional analyses to control for scanner platform. Although a parietal ROI exhibited group differences in developmental trajectories between groups at 4 months, these effects were no longer significant at 1 year or when additional analyses were conducted at 4 months to correct for scanner platform.

The neurodevelopmental literature has indicated that cortical myelination predominantly occurs during late childhood and adolescence, whereas white matter myelination occurs in early childhood (Corrigan et al., 2021, Deoni et al., 2012). The parietal lobe is well-known to have a strong correlation between age and GM myelination during late childhood and adolescence (Corrigan et al., 2021, Grydeland et al., 2013), which was replicated in the current study. However, there were no significant group differences in cortical myelination at approximately 1-week post-injury between pmTBI and HC. Although the HC group exhibited a significant increase in cortical GM myelination within the parietal lobes between V1 and V2 (4-month interval), this pattern was absent within the pmTBI group. Moreover, there were no significant group differences in parietal myelination at 1-year post-injury or at 4 months during additional sensitivity analyses with the ComBat algorithm, suggesting that any putative changes in parietal myelination as a result of injury were minimal. The current study employed a relatively large N and a statistically powerful prospective design, which collectively should have maximized the sensitivity to detect injury-related changes in GM myelin content following pmTBI.

We also examined for potential moderating effects associated with biological sex on injury-related effects, which were not present at current sample sizes. In contrast to previous findings (Corrigan et al., 2021, Norbom et al., 2020), increased cortical myelin content was observed for females relative to males. Other studies have also demonstrated that females reach peak GM volume at an earlier age (Lenroot et al., 2007) as well as increased cortical thickness (Goldstein et al., 2001, Leonard et al., 2008, Luders et al., 2006, Sowell et al., 2007). Therefore, additional work is required to better understand sexual dimorphisms related to corticial myelin content during neurodevelopment.

Previous preclinical (Mierzwa et al., 2015; Molina et al., 2020) and clinical (Jurick et al., 2018, Russell-Schulz et al., 2021, Spader et al., 2018, Wright et al., 2016, Yoo et al., 2022) studies on the effects of mTBI on myelin content have been varied and largely focused on collegiate (Spader et al., 2018, Wright et al., 2016) or adult (18–60) (Jurick et al., 2018, Russell-Schulz et al., 2021, Yoo et al., 2022) cohorts. Previous adult studies have also focused on either WM (Jurick et al., 2018, Russell-Schulz et al., 2021, Spader et al., 2018, Wright et al., 2016) or both WM and GM (Yoo et al., 2022) regions, with no publications on cortical myelination in children and adolescents following pmTBI to our knowledge. Wright et al. assessed concussions of collegiate hockey players and found that WM myelin content was reduced at 2 weeks post injury relative to the players’ baseline, but recovered by 2 months (Wright et al., 2016) supporting evidence of typical remyelination post-injury. In contrast, Spader et al. reported increased myelin content in collegiate football/rubgy players 72 h and 3 months, which was interpreted to be indicative of both typical and “exuberant” remyelination (Spader et al., 2018). Another study focusing on veterans found no differences in WM myelin content post mTBI using a traditional ROI approach years post-injury (Jurick et al., 2018). Other studies reported reduced WM myelin content in adult (18–60) mTBI patients when compared to HC either 1–5 months (Yoo et al., 2022) or 5 months – 6 years (Russell-Schulz et al., 2021) post injury, but no difference in GM myelin content (Yoo et al., 2022).

Previous adult mTBI studies used myelin water imaging (Russell-Schulz et al., 2021, Wright et al., 2016) or multicomponent Driven Equilibrium Single Pulse Observation (Jurick et al., 2018, Spader et al., 2018) rather than the T_1_w/T_2_w ratio method used in the current study to quantify myelin content. Debate remains about the optimal method for the *in vivo* quantification of myelin content for grey or white matter, with each approach having strengths and limitations (Corrigan et al., 2021, Lazari and Lipp, 2021, MacKay and Laule, 2016, Shafee et al., 2015, Shams et al., 2019, van der Weijden et al., 2021). The T_1_w/T_2_w ratio method is increasingly popular due to the common acquisition of both T_1_ and T_2_ sequences during imaging studies (Glasser et al., 2014, Glasser and Van Essen, 2011) coupled with relatively short acquisition times, robustness to movement artifacts (Shams et al., 2019), high spatial resolution, and high test–retest reliability (Arshad et al., 2017). The T_1_w/T_2_w ratio exhibits strong agreement with other MR-based GM myelin estimation methods (Hagiwara et al., 2018, Shams et al., 2019) as well as histology (Glasser and Van Essen, 2011). In contrast, methodological concerns have been raised in regards to the accuracy of the T_1_w/T_2_w ratio method for evaluating WM myelin content based on imaging (Arshad et al., 2017, Hagiwara et al., 2018, van der Weijden et al., 2021) and histological (Hagiwara et al., 2018, Sandrone et al., 2023) evidence. Thus, additional studies are needed to examine effects associated with potential methodological differences for *in vivo* myelin quantification to potentially explain discrepancies observed between current and previous results.

The precentral and postcentral gyrus exhibited the highest evidence of increased myelination as a function of age, consistent with the current understanding of typical myelination trajectories (Grydeland et al., 2019). The magnitude of the correlation was notably lower within the central sulcus, which is one of the first sulci to appear during cortical gyrification due to the rapid developmental integration of motor and somatosensory information during infancy and early childhood (Gajawelli et al., 2021). To our knowledge, this is one of the first studies to examine longitudinal differences in myelin content across the entire cortical mantle as a function of neurodevelopment. Interestingly, developmental changes in cortical myelination were greater in the left relative to right hemisphere as a main effect of study visit, as well as for the posterior parietal cortex region of interest. Previous cross-sectional studies have suggested that cortical myelination trajectories are approximately equivalent across the two hemispheres in late childhood and adolescence (Grydeland et al., 2013, Kwon et al., 2020), or adopted a region of interest approach that would not be sensitive to hemispheric differences (Kwon et al., 2020). In contrast, our longitudinal, large-N study was optimally designed to capture typical neurodevelopmental changes within individual participants even across relatively small periods of time. We hypothesize that the more rapid increase in left hemisphere myelin content may be secondary to this hemisphere’s greater contribution to language and mathematics, which is still rapidly improving during late childhood and adolescent age (Holland et al., 2001, Rivera et al., 2005). An alternative hypothesis is that the right hemisphere myelinates more quickly than the left hemisphere from a developmental perspective, and thus undergoes less rapid changes during adolescence. Additional research across a larger age range is therefore needed to investigate individual host factors that affect myelin development between hemispheres.

Multidimensional clinical assessments (cognitive, physical, and emotional) revealed varying recovery patterns, with some symptoms improving by 4 months post-injury (pain, headache, attention, and executive function), while others persisting to 1 year (sleep, functional outcomes, behavior, and long-term memory). A recent *meta*-analysis found that sleep disturbances remained higher in pmTBI than the general population several months post-injury (Djukic et al., 2022), which could lead to impaired functional outcomes including memory consolidation as observed in our cohort (Tononi and Cirelli, 2014). These findings highlight the need for comprehensive, multidimensional clinical assessments to evaluate the developmental risks and long-term neuropsychological changes that can occur in a subset of pmTBI patients, which can provide a better insight into the underlying injury mechanisms.

There are several limitations to the study. Foremost, two different scanner platforms and two different T_2_ sequences were utilized across the entire study cohort, which resulted in different T_1_w/T_2_w ratios (Supplementary Fig. S4), a known issue with the methodology (Glasser and Van Essen, 2011). Although we attempted to control for scanner/sequence effects in our models through multiple statistical methods and sensitivity analyses (i.e., both residualization as well as harmonization), it is possible that this increased variance reduced sensitivity to longitudinal group effects. Furthermore, there was also a 11% loss in data for V3 analysis secondary to changes in scanner platforms and software versions (i.e., purposeful non-scanning of participants due to known differences in scanner hardware). Additionally, although our 4-month retention rates were relatively high (82% and 94% retention for pmTBI and HC respectively), retention decreased below the recommended 70% levels for the 1-year follow up (69% and 82% retention for pmTBI and HC respectively). Both primary (age) and Supplemental (sex and self-reported pubertal status) analyses examined for any potential moderating effects of neurodevelopment on injury-related effects in myelin content. However, even though the current study represents the largest pediatric sample examing myelin content to date, it may still have been underpowered to detect complex interactions between trauma, neurodevelopment and sex-related changes. Finally, the current study compared data separately from ∼7 days to ∼4 months and ∼1 year post-injury to investigate both short- and long-term effects associated with trauma, which precluded the use of a single longitudinal pipeline (Reuter et al., 2012).

In summary, our findings provide evidence of rapid (i.e., occurring over a 4-month period of time between V1 and V2) increases in cortical myelination during middle childhood and adolescence, which may occur in a hemisphere specific fashion. There was minimal evidence suggesting that pmTBI resulted in consistent, long-term changes in GM myelin content or GM myelination as a function of neurodevelopment or due to sex-related differences. Additional studies are needed to confirm these findings, as well as more appropriate MRI methods to estimate trauma related changes in WM myelin content.

## CRediT authorship contribution statement

**Jessica R. McQuaid:** Writing – original draft, Formal analysis, Conceptualization. **Tracey V. Wick:** Writing – original draft, Visualization, Formal analysis, Data curation, Conceptualization. **Josef Ling:** Software, Methodology, Formal analysis. **Andrew B. Dodd:** Writing – review & editing, Formal analysis. **Divyasree Sasi Kumar:** Writing – review & editing, Formal analysis. **Upasana Nathaniel:** Writing – review & editing, Formal analysis. **Samuel D. Miller:** Writing – review & editing, Formal analysis. **Vadim Zotev:** Writing – review & editing. **Harm J. van der Horn:** Writing – review & editing. **John P. Phillips:** Writing – review & editing. **Richard A. Campbell:** Writing – review & editing. **Robert E. Sapien:** Writing – review & editing. **Timothy B. Meier:** Writing – review & editing. **Andrew R. Mayer:** Writing – original draft, Supervision, Resources, Investigation, Funding acquisition, Formal analysis, Conceptualization.

## Declaration of Competing Interest

The authors declare that they have no known competing financial interests or personal relationships that could have appeared to influence the work reported in this paper.

## Data Availability

The data that support the findings of this study will be openly available in FITBIR at fitbir.nih.gov upon the conclusion of the study, reference number FITBIR-STUDY0000339.
