# Supplementary material for: A one year longitudinal study of cortical myelination changes following pediatric mild traumatic brain injury
Source: Neuroimage Clin. 2025 Jun 30;48:103837. doi: 10.1016/j.nicl.2025.103837 (PMC12275868; doi:10.1016/j.nicl.2025.103837)
Supplement: Supplementary Data 1 [file mmc1.docx]

**Supplemental Materials**

**Methods**

*Participants*

Quality assurance for the myelin maps was completed through careful visual inspection by a minimum of two trained researchers individually for each visit. Outlier analysis was performed separately for each scanner platform (Siemens Tim Trio v. Prisma Fit) using the smoothed myelin maps. Myelin maps with outlying vertices totaling above 5% were flagged for visual inspection by a minimum of two trained researchers. Myelin maps with a total of outlying vertices above 10% or participants with a note regarding issues collecting data (recorded by RA administering appointment at the time of appointment) were removed from the analysis. Participants with incomplete data (i.e., acquisition of T2 missing) or excluded during quality assurance steps remained eligible for following visit analyses pending other exclusionary criteria and quality assurance.

From the recruited 247 pmTBI and 200 HC, 4 participants (2 pmTBI) were excluded for MRI contraindications, 6 participants (4 pmTBI) for incomplete data (i.e., acquisition of T2 missing), 33 participants (18 pmTBI) for quality assurance, and 7 participants (6 pmTBI) following outlier analysis. The final V1 sample used in analysis included 217 pmTBI (91 females; 14.3 ±2.9 years old; 7.5±2.1 days post-injury) and 180 HC (85 females; age 14.2 ±2.9 years old).

Attrition occurred for 44 pmTBI (82.0% retention) and 12 HC (93.9 % retention) between V1 and V2. A subset of 4 pmTBI participants were unable to return for V2 only and returned for V3 visit. One pmTBI participant was lost to follow-up due to COVID-19 pandemic restrictions. This resulted in 200 pmTBI and 186 HC eligible for imaging data during V2.

At V2, 3 participants (2 pmTBI) declined the MRI, 7 participants (6 pmTBI) did not complete an MRI due to the MRI upgrade, 5 pmTBI participants did not complete an MRI due to new MRI contraindications, 5 participants (3 pmTBI) did not complete an MRI due to new braces, 2 HC participants had positive drug screens, 2 HC participants were diagnosed with a new psychiatric disorder between V1 and V2, 1 HC participant was disenrolled from the study due to unreliable arrival at V1 (multiple rescheduling of V1 and tardiness to appointment), 8 participants (4 pmTBI) had incomplete data, 30 participants (14 pmTBI) were excluded following visual QA, and 4 participants (3 pmTBI) were excluded following outlier analysis. The final V2 sample used in analysis included 159 pmTBI (69 females; 132.8±15.0 days post-injury; 125.2±14.8 days between visits) and 156 HC (69 females; 129.7±19.6 days between visits).

Attrition occurred for 16 pmTBI (91.3% retention) and 15 HC (91.5% retention) between V2 and V3. While retention rates between V1 and V2, as well as between V1 and V3, were relatively high based on exclusion criteria, by V3 there was a significant loss in sample size with only 68.6% of recruited pmTBI and 81.8% recruited HC eligible for V3 data analysis before any exclusions or data assurance at V3. 3 pmTBI participants were lost to follow-up due to COVID-19 pandemic restrictions. This resulted in 165 pmTBI and 162 HC eligible for imaging data during their V3 visit.

At V3, 36 participants (10 pmTBI) did not complete an MRI due to the MRI upgrade, 6 participants (4 pmTBI) did not complete an MRI due to new MRI contraindications, 10 participants (6 pmTBI) did not complete an MRI due to new braces, 2 participants (1 pmTBI) diagnosed with a new medical condition between V2 and V3, 4 participants (1 pmTBI) had a positive drug screen, 4 participants (1 pmTBI) were diagnosed with a new psychiatric disorder between V2 and V3. 4 pmTBI participants had incomplete data, 52 participants (22 pmTBI) were excluded following visual QA which included 18 participants (5 pmTBI) who were run on a different MRI software with poor T2 quality, and 3 pmTBI participants were excluded following outlier analysis. The final V3 sample used in the analysis included 113 pmTBI (45.7% of recruited pmTBI; 51 females; 365.9±36.1 days post-injury; 361.1±31.4 days between visits) and 93 HC (46.5% of recruited HC; 43 females; 363.4±32.1 days between visits).

*Clinical analyses*

All clinical and cognitive results were Bonferroni corrected based on primary versus secondary measures (Supplementary Table 1). Primary clinical measures included three domains (PCS burden, pediatric quality of life, and self-reported behavioral disturbances) with a Bonferroni correction of *p* < 0.0167 (p=0.050/3), and secondary clinical measures included seven domains (pain scale, headache, anxiety, sleep, global outcome, behavioral and emotional difficulties, and depression) with a Bonferroni correction of *p* < 0.007 (p=0.050/7). Primary cognitive measures included two domains (attention and processing speed) with a Bonferroni correction of *p* < 0.025 (p=0.05/2), and secondary cognitive measures included three domains (executive function, long-term memory, and working memory) with a Bonferroni correction of *p* < 0.0167 (p=0.050/3).

*Statistical analyses*

Sensitivity analyses were conducted for clinical and cognitive measures using general estimating equations (GEE) using normal distributions based on information criterion results separately for each Group (HC and pmTBI) and each follow-up visit (V2 and V3). Sensitivity analysis of cortical myelin content was completed using the AFNI program 3dLME. Analysis compared V1 metrics (clinical and cognitive measures, ROI cortical myelin content, whole-brain cortical myelin content) of participants who returned for V2/V3 to participants who did not return for V2/V3.

Additional demographic analysis was completed for age (unit=months) separately for each visit (V1, V2, V3) comparing Group (pmTBI vs. HC). The participant age was corrected for V2 and V3 analysis based on the participants' days between visits (DBV) to obtain the most accurate estimation of age. Analysis comparing DBV between Group was completed for V2 and V3 separately.

*Image Acquisition*

Image acquisition was performed using either 3T Siemens Tim Trio Scanner or 3T Prisma Fit VE11 with a 32-channel head coil. Large scanner effects were seen between the Tim Trio and Prisma Fit scanner (Figure S4). For anatomical reference for participants scanned using the 3T Siemens Tim Trio scanner, a high-resolution 5-echo Magnetization Prepared Rapid Acquisition Gradient Echo (MPRAGE) T_1_–weighted [repetition time (TR)=2530 ms; echo times (TE)=1.64, 3.5, 5.36, 7.22, 9.08 ms; inversion time (TI)=1200 ms; flip angle=7°; number of excitations (NEX)=1; slice thickness=1 mm; field of view (FOV)=256 mm; matrix size=256 × 256; isotropic voxels=1 mm^3^] was used. In addition, T_2_-weighted sequence [TR=15500 ms; TE=77 ms; flip angle=155°; NEX=1; slice thickness=1.5mm; FOV=220 mm; matrix size=192×192; voxel size=1.15 × 1.1.5 × 1.5 mm], susceptibility-weighted [TR = 28 ms; TE = 20.0 ms; flip angle = 15°; NEX = 1; slice thickness = 1.5 mm; FOV = 192 × 256; matrix size = 192 × 256; voxel size = 1.00 × 1.00 × 1.50 mm] and fluid-attenuated inversion recovery (FLAIR) data were collected using the following parameters [TR = 10380 ms; TE = 88.0 ms; TI = 2500 ms; flip angle = 140; NEX = 1; slice thickness = 3 mm; FOV = 256; matrix size = 320×320; 50 interleaved slices; 0.80×0.80×3.00 mm voxels] were used.

For anatomical reference for participants scanned using the 3T Prisma Fit VE11, a high-resolution 5-echo Magnetization Prepared Rapid Acquisition Gradient Echo (MPRAGE) T_1_–weighted [repetition time (TR)=2530 ms; echo times (TE)=1.61, 3.47, 5.33, 7.19, 9.05 ms; inversion time (TI)=1200 ms; flip angle=7°; number of excitations (NEX)=1; slice thickness=1 mm; field of view (FOV)=256 mm; matrix size=256 × 256; isotropic voxels=1 mm^3^]. In addition, T_2_-weighted sequence [TR=3200 ms; TE=428 ms; flip angle=120°; NEX=1; slice thickness=1mm; FOV=256 mm; matrix size=256 × 256; voxel size=1.0 × 1.0 × 1.0 mm], FLAIR [TR=10380 ms; TE=89 ms; TI=2500 ms; flip angle=160°; NEX=1; slice thickness=3mm; FOV=256 mm; matrix size=320 × 320; 50 interleaved slices; voxel size=0.8 × 0.8 × 3 mm], and susceptibility-weighted [TR = 28 ms; TE = 20.0 ms; flip angle = 15°; NEX = 1; slice thickness = 1.5 mm; FOV = 192 × 256; matrix size = 192 × 256; 88 interleaved slices; voxel size = 1.00 × 1.00 × 1.50 mm].

**Supplemental Results**

*Cortical Myelin Results*

When comparing V1 and V2 cortical myelin content, a significant main effect was observed for Visit in the left frontal middle sulcus (59.09 mm^2^), orbital part of the inferior frontal gyrus (54.64 mm^2^), superior precentral sulcus (668.36 mm^2^), precentral gyrus (82.88 mm^2^), superior frontal gyrus (284.46 mm^2^ and 111.29 mm^2^), marginal branch of the cingulate sulcus (111.61 mm^2^), central sulcus (55.80 mm^2^), postcentral sulcus (59.52 mm^2^), intraparietal and transverse parietal sulcus (8596.16 mm^2^), middle temporal gyrus (296.67 mm^2^ and 76.06 mm^2^), inferior temporal sulcus (49.52 mm^2^), inferior temporal gyrus (73.02 mm^2^), inferior occipital gyrus and sulcus (95.62 mm^2^), and middle occipital gyrus (91.12 mm^2^; Figure S1). Significant effects of visit were also observed within the right intraparietal and transverse parietal sulcus (105.39 mm^2^), superior frontal gyrus (140.9 mm^2^), central sulcus (69.13 mm^2^), and precuneus (269.02 mm^2^ and 144.07 mm^2^; Figure S1). The increase in cortical myelination between V1 to V2 was greatest in the left parietal region.

When comparing V1 and V3 cortical myelin content, a significant main effect of increased myelin content was observed for V3 in the left superior frontal gyrus (34.92 mm^2^), precentral gyrus (50.75 mm^2^), middle temporal gyrus (195.18 mm^2^), superior parietal gyrus (8988.92 mm^2^), and middle occipital gyrus (212.03 mm^2^ and 164.34 mm^2^; Figure S1). Increased myelin content was also observed within the right central sulcus (716.96 mm^2^), superior parietal gyrus (64.48 mm^2^), and superior occipital gyrus (256.32 mm^2^; Figure S1). In contrast, the left cingulate sulcus and intracingulate sulcus exhibited decreased myelin content at V3 (V1>V3, 79.83 mm^2^; Figure S1).

Sepearate 2×2×2 [Group (pmTBI v. HC) × Visit (V1 v. other) × Sex (male v. female)] LME models with scanner and pubertal status as covariate were performed to examine any significant potential moderating effect of Sex or pubertal stauts across both models (i.e., V1 vs V2 and V1 vs. V3). However, the main effect of Group and the Group × Visit interaction were mostly unchanged. A significant main effect of increased myelin content was observed for females relative to males both hemispheres of the brain (Figure S3).

**Table S1:** Primary and secondary clinical and cognitive measures.

| **Instrument** | **Measured domain** | **Status** | **Rater** | **Visit** |
| --- | --- | --- | --- | --- |
| **Demographics** | | | | |
| NewMAP TBI | Self-reported TBI history | Secondary | C & P | R, V1, V2, V3 |
| Tanner Stage of Development | Pubertal development | Secondary | C | V1, V2 & V3 |
| ASSIST | Use of alcohol and other drugs | Secondary | C | V1, V2 & V3 |
| BSI-18 | Parental psychopathology | Secondary | P | V1, V2 & V3 |
| **Clinical Domain** | | | | |
| PCSI | Post-concussive symptoms | Primary | C | R, V1, V2 & V3 |
| PROMIS Sleep | Sleep disturbance | Secondary | C | R, V1, V2 & V3 |
| PROMIS Anxiety | Anxiety symptoms | Secondary | C | R, V1, V2 & V3 |
| PROMIS Depression | Depressive symptoms | Secondary | C | R, V1, V2 & V3 |
| Pain scale | Pain | Secondary | C | R, V1, V2 & V3 |
| HIT-6 | Headache symptoms | Secondary | C | R, V1, V2 & V3 |
| CBQ | Family conflict | Primary | C | R, V1, V2 & V3 |
| SDQ | Behavioral screening for psychological attributes | Secondary | P | R, V2 & V3 |
| PedsQL | Health-related quality of life | Primary | C | R & V2 & V3 |
| GOS-E | Functional outcome | Secondary | C & P | V1, V2 & V3 |
| **Cognitive Domain** | | | | |
| TOMMe10 | Measure of effort | Secondary | C | V1, V2 & V3 |
| WRAT-4 | Premorbid reading ability | Secondary | C | V1, V2 & V3 |
| DKEFS Color-Word interference Cond 1-3 | Attention | Primary | C | V1, V2 & V3 |
| WAIS-IV/WISC-V Coding and Symbol Search | Processing speed | Primary | C | V1, V2 & V3 |
| WISC-V/WAIS-IV Digit Span Backwards | Working memory | Secondary | C | V1, V2 & V3 |
| DKEFS Trail Making Test Cond 2 and 4, Verbal Fluency, Color-Word interference Cond 4 | Executive function | Secondary | C | V1, V2 & V3 |
| HVLT Delayed Recall | Long-Term Memory Recall | Secondary | C | V1, V2 & V3 |

Notes: Instrument-- NewMAP TBI: New Mexico Assessment of Pediatric TBI, ASSIST: The Alcohol, Smoking and Substance Involvement Screening Test, BSI: Brief Symptom Inventory-18, PCSI: Post-Concussion Symptom Inventory, PROMIS: Patient-Reported Outcomes Measurement Information System, HIT-6: Headache Impact Test, CBQ: Conflict Behavior Questionnaire, SDQ: Strengths and Difficulties Questionnaire, PedsQL: Pediatric Quality of Life Inventory, GOS-E: Glasgow Outcome Scale Extended, TOMMe10: Test of Memory Malingering, WRAT-4: Wide Range Achievement Test, DKEFS: Delis-Kaplan Executive Function System, WAIS-IV: Wechsler Adult Intelligence Scale–IV, WISC-V: Wechsler Intelligence Scale for Children–V, HVLT: Hopkins Verbal Learning Test; Rater—C: child, P: parent; Visit—R: retrospective,.

**Table S2**: ROI Regions

| Destrieux Index Number | Region Name |
| --- | --- |
| 25 | Angular gyrus |
| 26 | Supramarginal gyrus |
| 27 | Superior parietal lobule |
| 30 | Precuneus |
| 55 | Sulcus intermedius primus |
| 56 | Intraparietal sulcus & transverse parietal sulci |
| 71 | Subparietal sulcus |

Notes: These are the seven regions used for the parietal ROI. All regions were taken from the Destrieux Atlas with the indicated index number

**Table S3**: Demographics and injury characteristic data.

|  | V1 pmTBI  (N=217) | V1 HC  (N=180) |
| --- | --- | --- |
| Age | 14(12-16) | 14(11-16) |
| Sex (% Female) | 41.9% | 47.2% |
| Tanner Stage of Development | 4(3-4) | 4(2-4) |
| Parent BSI-18^a^ | 3(1-7) | 1(0-4) |
| pmTBI Hx^a^ | 18.0% | 7.2% |
| **Injury Characteristics** |  |  |
| Loss of Consciousness | 48.4% | - |
| Post-Traumatic Amnesia | 39.2% | - |
| **Mechanism of Injury** |  |  |
| Struck by Object | 13.4% | - |
| Struck by Person | 20.3% | - |
| Fall | 30.0% | - |
| MVC | 29.0% | - |
| Assault | 3.2% | - |
| Bicycle-related | 3.2% | - |
| Other | 0.9% | - |
| **Sport or Recreation Related** | 55.3% | - |

Notes: HC=healthy control; pmTBI=pediatric mild traumatic brain injury; BSI=Brief Symptom Inventory-18; MVC=motor vehicle crash; Hx = history. Data are formatted at mean ± standard deviation or median (interquartile range) based on distribution properties. ^a^= Group main effect


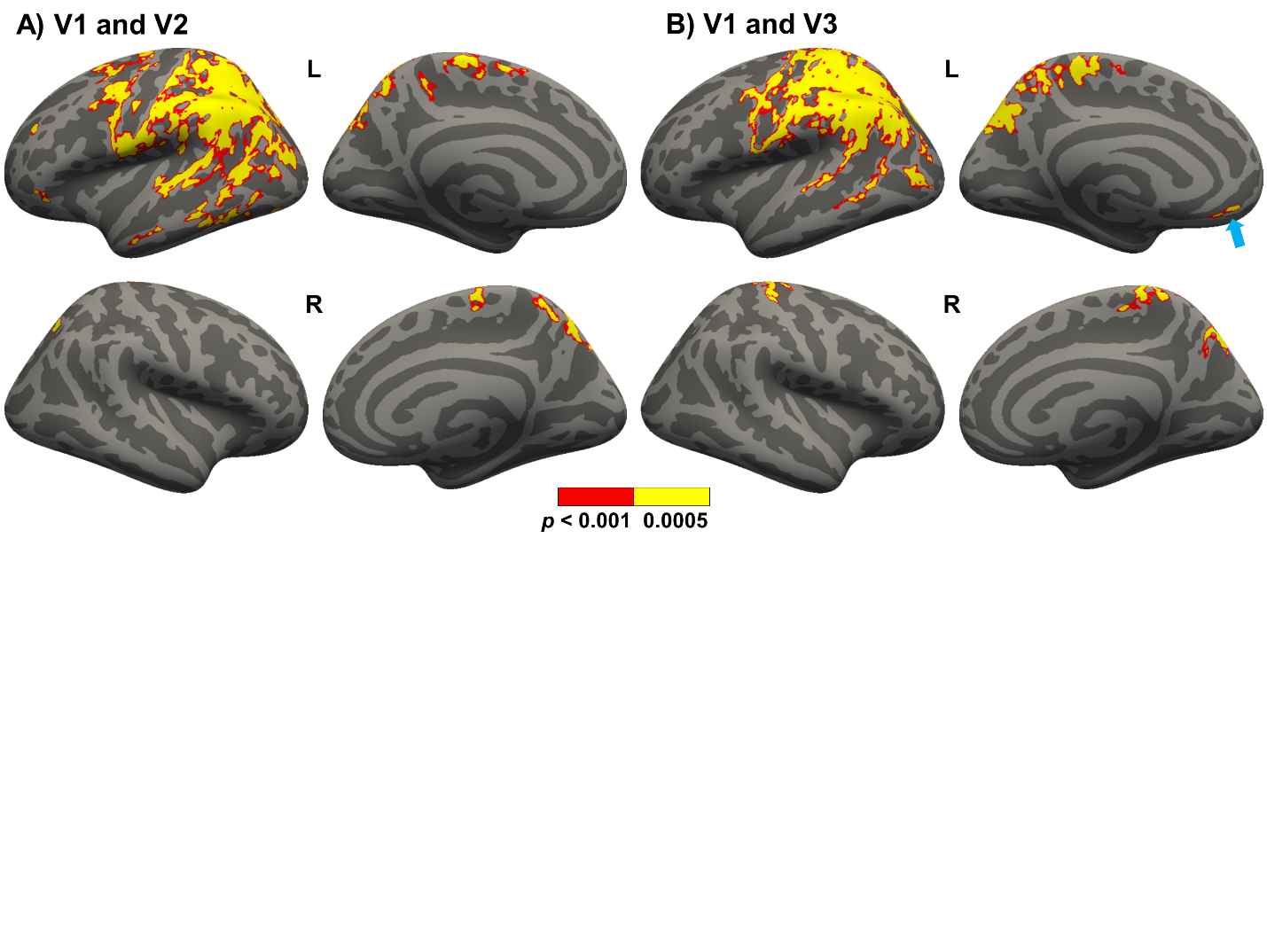


**Figure S1.** Vertex-wise cortical myelin results for the main effect of Visit. Regions within the left (L) and right (R) hemispheres that showed significant changes in myelin content across both groups of participants at visit 2 (V2; Panel A) or visit 3 (V3; Panel B) relative to visit 1 (V1). All regions demonstrated a significant increase in cortical myelin content between visits with the exception of the left cingulate and intracingulate sulcus as indicated by the blue arrow (Panel B). Significant results are depicted for p<0.001 (red) and p<0.0005 (yellow) thresholds. Increases in myelin content were greatest within the parietal lobe as a function of study visit, with a notable lack of myelination within the central sulcus.


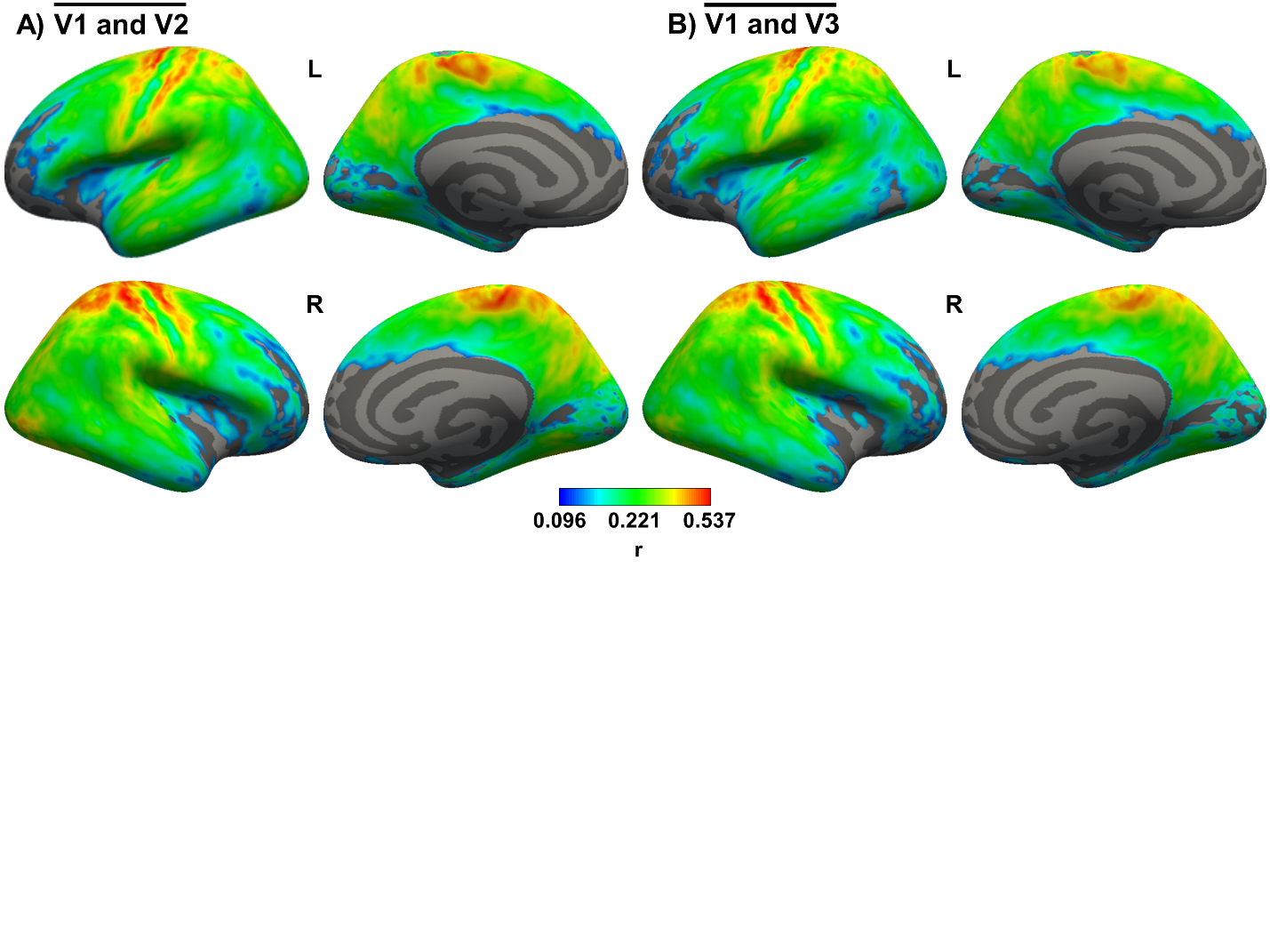


**Figure S2.** Vertex-wise results for the association between cortical myelin and chronological age. Regions demonstrating a significant positive relationship between cortical myelin content and age when comparing the mean of Visits 1 (V1) and 2 (V2; Panel A) as well as Visits 1 and 3 (V3; Panel B). For display, data were averaged across both visits (denoted by thick black line), and the Pearson correlation (r) value projected onto the cortical mantle (range 0.096 to 0.537). The strongest relationships between age and myelin content were observed in the precentral gyrus, postcentral gyrus, paracentral gyrus and sulcus, and superior parietal gyrus. The relationship between myelin and chronological age was notably lower within the central sulcus.


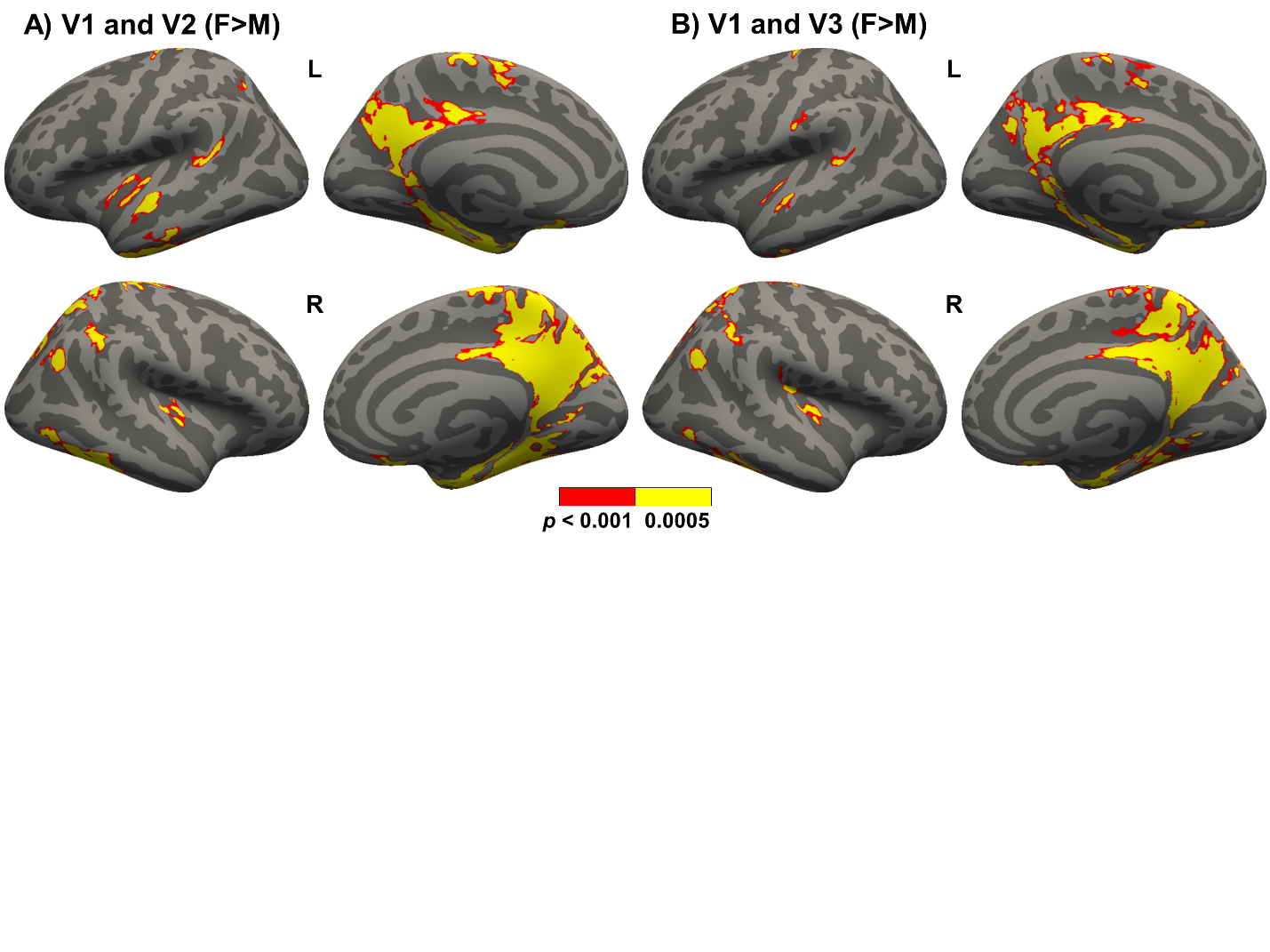


**Figure S3.** Vertex-wise cortical myelin results for the main effect of Sex. Regions within the left (L) and right (R) hemispheres that showed significant increased myelin content in females (F) relative to males (M) across study visit (V) comparisons. Significant results are depicted for p<0.001 (red) and p<0.0005 (yellow) thresholds.


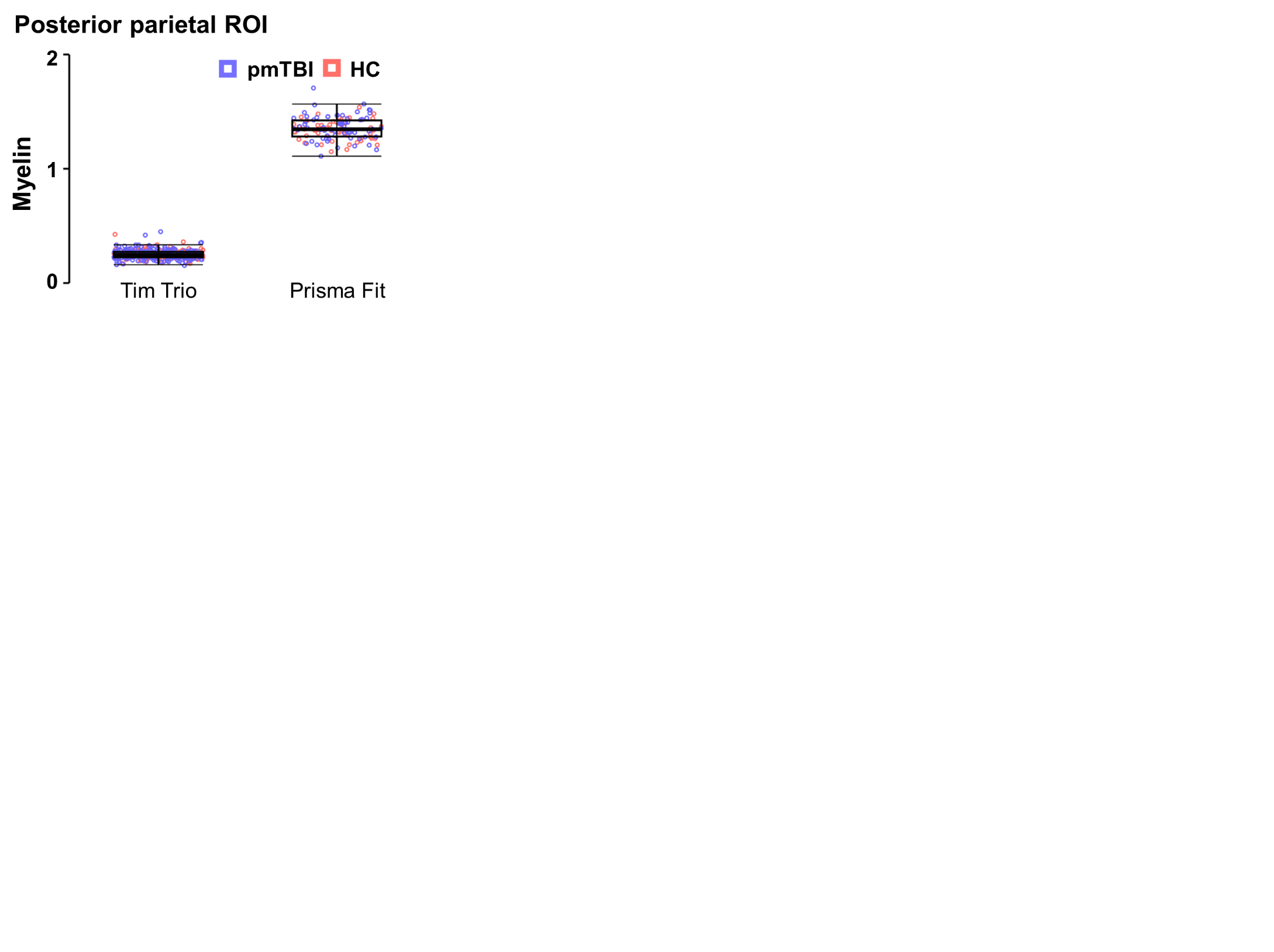


**Figure S4.** Differences in cortical myelin content across the two scanner (Tim Trio and Prisma Fit) platforms and sequences within the parietal ROI. The T_1_w/T_2_w ratio method estimated a much higher myelin content in the Prisma Fit than the Tim Trio, leading to large scanner effects for all analyses.
